# Positionally-conserved but sequence-diverged: identification of long non-coding RNAs in the Brassicaceae and Cleomaceae

**DOI:** 10.1186/s12870-015-0603-5

**Published:** 2015-09-11

**Authors:** Setareh Mohammadin, Patrick P. Edger, J. Chris Pires, Michael Eric Schranz

**Affiliations:** Biosystematics, Plant Science Group, Wageningen University, Droevendaalsesteeg 1, 6708 PB Wageningen, The Netherlands; Division of Biological Sciences, University of Missouri, Columbia, MO 65211 USA

## Abstract

**Background:**

Long non-coding RNAs (LncRNAs) have been identified as gene regulatory elements that influence the transcription of their neighbouring protein-coding genes. The discovery of LncRNAs in animals has stimulated genome-wide scans for these elements across plant genomes. Recently, 6480 LincRNAs were putatively identified in *Arabidopsis thaliana* (Brassicaceae), however there is limited information on their conservation.

**Results:**

Using a phylogenomics approach, we assessed the positional and sequence conservation of these LncRNAs by analyzing the genomes of the basal Brassicaceae species *Aethionema arabicum* and *Tarenaya hassleriana* of the sister-family Cleomaceae. Furthermore, we generated transcriptomes for another three *Aethionema* species and one other Cleomaceae species to validate their transcriptional activity. We show that a subset of LncRNAs are highly diverged at the nucleotide level, but conserved by position (syntenic). Positionally conserved LncRNAs that are expressed neighbour important developmental and physiological genes. Interestingly, >65 % of the positionally conserved LncRNAs are located within 2.5 Mb of telomeres in *Arabidopsis thaliana* chromosomes.

**Conclusion:**

These results highlight the importance of analysing not only sequence conservation, but also positional conservation of non-coding genetic elements in plants including LncRNAs.

**Electronic supplementary material:**

The online version of this article (doi:10.1186/s12870-015-0603-5) contains supplementary material, which is available to authorized users.

## Background

Gene regulatory transcripts are crucial in expressing or repressing protein coding genes. For example, gene repression in plants can be maintained by microRNAs (miRNAs, 19-22 nt long) and small interfering RNAs (siRNAs, 23-24 nt long). While miRNAs are mainly involved with the post-transcriptional gene repression, siRNAs are also involved pre-transcriptional gene repression by the *de novo* deposition of chromatin marks [1]. A new category of RNA dependent gene regulators are Long non-coding RNAs (LncRNAs, longer than 200 nt, ORF smaller than 100 amino acids) that can act in the course of pre-transcriptional repression of gene-expression [2–4].

Long non-coding RNAs can silence genes by acting as a sequence-specific template for chromatin or associate with downstream proteins [3] and are transcribed from the intergenic (long intergenic non-coding RNAs = LincRNAs), intronic or anti-sense regions [5, 6]. Recently it has been shown for the LncRNAs COOLAIR in *Arabidopsis thaliana* [7, 8] and for the rice LncRNA LDMAR [9, 10] how they influence the expression of phenotypically important regulatory genes. COOLAIR (cold induced long antisense intragenic RNA) is transcribed from the *Flowering Locus C (FLC)* and accelerates the transcriptional repression of *FLC* during cold by reducing the gene activating chromatin mark H3K36me3 [7]. In parallel, the gene silencing chromatin mark H3K27me3 is accumulating at the intragenic *FLC* nucleation site by a Polycomb-directed process [7]. Thus, LncRNAs COOLAIR contributes to the induction of flowering after vernalization. The mutant rice 58S has infertile pollen under long days, while the pollen are variably fertile under short days. Ding et al. [9] found that when LncRNA LDMAR is overexpressed in 58S rice recovers fertility under long days. The transcription of LDMAR in 58S is controlled by a negative feedback loop with a siRNA called Psi-LDMAR. Psi-LDMAR is transcribed from the promoter region of LDMAR. Psi-LDMAR induces RNA dependent DNA methylation; this leads to a reduction in the transcription of LDMAR and hence reduces the fertility of 58S under long days [10]. These recent discoveries of plant LncRNAs highlight their influence on important fitness traits, e.g. male sterility (LDMAR) and flowering time (COLDAIR, COOLAIR, IPS1) [8, 9]. The influence of LncRNAs on regulating chromatin structure shows their involvement to permit plants to respond to environmental cues [3].

LncRNAs have also been identified and studied in other plants, including *Zea mays, Triticum aestivum* and *Oryza sativa* [11–13]. These genome-wide identifications of LncRNAs were done using existing EST sequences, full-length cDNA databases and/or full genome tiling microarrays [11–13]. Li et al. [11] found more than 20,000 putative LncRNAs in rice; although >90 % were assigned to being small RNA precursors. A similar result was found in *Zea mays* where ~60 % of the LncRNAs are probably small RNAs precursors [14]. About 40 % of the rice non-exonic transcription active regions seem to be potential non-coding RNAs [11]. Liu et al. [5] found 6480 LincRNAs in the model plant *Arabidopsis thaliana* (Brassicaceae). Some of these putative L(i)ncRNAs were further validated with expression pattern analyses, custom microarrays and RNA-seq [5, 11–13]. However all these studies have thus far relied on analyses of only a single species.

Inter-species genome-wide comparisons have shown that protein-coding genes are not only conserved by sequence, but can also be conserved by their position in the genome (e.g. synteny) [15]. The conservation of a genomic position over different phylogenetic scales can indicate that the position of a given gene is under strong purifying selection [16]. The genome-wide duplication history of *Arabidopsis thaliana* (Brassicaceae) was revealed by the identification and analyses of collinear duplicated blocks that arose from multiple ancient whole genome duplications [17]. Recently, the genome of *Aethionema arabicum*, a member of the Tribe Aethionemeae in the earliest diverging lineage of the Brassicaceae, was sequenced [18] as well as the genome of *Tarenaya hassleriana* of the Cleomaceae, the sister-family to the Brassicaceae [19]. The comparisons of these three genomes provide insights into which genes and intergenic regions may be conserved by position between Brassicaceae-Cleomaceae. However, the genome sequences are not enough to understand their potential functional significance. Hence it is also valuable to have transcriptome data to complement the genome data of species at evolutionary important positions to infer the positional conservation of regulatory transcripts including LncRNAs.

**Fig. 1:**
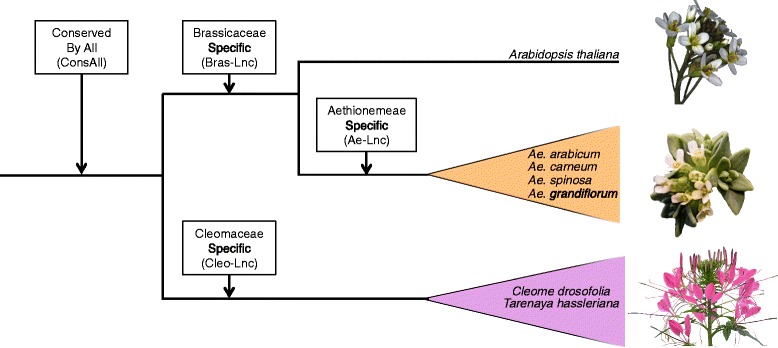
Simplified phylogeny of the Brassicaceae and Cleomaceae highlighting target species used to identify Long non-coding RNAs (LncRNAs). The boxes *above* the branches represent the studied lineages, their specificity at the sequence level and their abbreviations. Pictures show (from *top* to *bottom*) the inflorescences of *Arabidopsis thaliana, Aethionema arabicum* and *Tarenaya hassleriana*

Here we used the genomes of *Ae. arabicum*, *T. hassleriana* and *A. thaliana* in addition to our newly generated transcriptome data of four Aethionemeae and two Cleomaceae species to understand the conservation of LncRNAs in a phylogenomic context (Fig. 1). We not only analysed the nucleotide conservation of LncRNAs, but also whether or not they were conserved by genomic position. We found that of the LncRNAs that seem sequence-specific (e.g. lineage-specific) to the Cleomaceae, Brassicaceae or Aethionemeae, >25 % are conserved by position. This positional conservation could tell us more about the putative function of these LncRNAs, and the evolutionary importance of positional conservation of these genomic features.

## Results

### Sequence conservation

We identified LncRNAs in four Aethionemeae and two Cleomaceae species from transcriptome data. To assess the sequence conservation of these LncRNAs we used OrhtoMCL [20]. For the positional conservation we used the CoGe tools SynFind and GeVo [21].

We used a previous classification of LncRNAs in *Arabidopsis* [5]: 1) LincRNA if the transcriptional unit (TU) was ≥500 bp away from the nearest protein coding gene, regardless if on sense or antisense strand. 2) Gene Associated Transcriptional Unit (GATU) if the TU was within a 500 bp range of a protein-coding gene. 3) ‘TU encoding NAT’ if the TU was transcribed from the opposite strand than the sense strand of a protein coding gene. 4) miRNA precursors, which can have long transcripts as precursors.

We assessed whether the 6480 *A. thaliana* LincRNAs (Ath-Linc) assessed by [5] were conserved throughout the Brassicaceae and Cleomaceae (All-Lnc) with an OrthoMCL analysis; a cluster algorithm based on reciprocal best blast hits [20]. The analysis included Ath-Linc and the genomes of *Aethionema arabicum* and *Tarenaya hassleriana* (see Methods and Additional file 1: Figure S1 for details)*.* Because LncRNAs have a higher mutation rate than protein coding sequences [14, 22], the analysis was done using increasing sequence similarity cut-off values of ≥10 %, ≥20 % and ≥50 %. Out of the 6480 Ath-Lincs only eleven are conserved by all three species at the genomic level. Out of these eleven conserved Ath-Lincs, only nine are transcribed in all three species based on our RNA-seq data (see below) and the RNA-seq data of [5] (Additional file 2: Table S1 for the average transcript and ORF lengths of these LncRNAs). Conserved Ath-Lincs were blasted (local BlastN) against the NCBI-database to assess whether the sequences were conserved in other organisms. At3NC056191, with a sequence similarity of ≤20 % with the *Ae. arabicum* and *T. hassleriana* transcriptomes and genomes, was homologous in sequence to the 5.8S ribosomal RNA gene and internal transcribed spacer 2 to the oomycete *Albugo laibachii.* The genomically conserved At2NC003370, At4NC004390 and At4NC004390 were conserved across most land plants, including the bryophyte *Physcomitrella patens* (Additional file 3).

We defined a lineage-specific LncRNA that is shared at the nucleotide level by multiple species within our focal lineages (e.g. Brassicaceae, Aethionemeae or Cleomaceae), but not found in other lineages. There were fifteen Ath-Lincs that were specific only to the Brassicaceae (Bras-Lnc, see Fig. 1). To ascertain that the Ath-Lincs and their corresponding *Ae. arabicum* transcripts were restricted to the Brassicaceae we compared them against the NCBI and Phytozome databases using BlastN, BlastX and TblastX (see Methods and Additional file 1: Figure S1 for details and cut-off values). Of the fifteen Bras-Lncs, nine were transcribed by *Ae. arabicum* and/or *A. thaliana* (Additional file 4: Table S3 for the average transcript and ORF length of the *Ae. arabicum* transcripts).

To test for Aethionemeae specific LncRNAs (Ae-Lnc) we generated RNA-seq data for four Aethionemeae species: *Ae. arabicum, Ae. carneum, Ae. grandiflorum* and *Ae. spinosa.* We identified 15 LncRNAs Ae-Lncs that were ≥50 % similar in sequence between these four Aethionemeae species (see Methods and Additional file 5: Figure S2 for pipeline). These fifteen Ae-Lncs correspond to 15, 15, 16 and 20 transcripts in *Ae. arabicum, Ae. carneum, Ae. grandiflorum* and *Ae. spinosa* respectively (from the total of 19,037, 18,305, 48,609 and 60,772 predicted transcripts). The average ORF length (±SD) of the putative LncRNAs across all four species was 145.89 bp (±10.00 bp) with an average transcript length of 546.83 bp (±28.63 bp SD) (Additional file 6: Table S4 for species specific averages). The Ae-Lnc consisted of two GATUs, four TUs encoding NATs and nine LincRNAs (Additional file 3 and Additional file 7: Table S2). Two Ae-LncRNAs are micro-RNA precursors for ath-MIR403 and aly-MIR408 (MFE of −71.8 and −74.2 kcal/mol respectively). Although ath-MIR403 is not tissue specifically expressed, under hypoxic conditions it is more present in leaves and whole plants than in roots [23, 24]. The function and tissue specificity of aly-MIR408 is not known [25].

For the Cleomaceae-specific LncRNA (Cleo-Lnc), RNA-seq data of *Tarenaya hassleriana* and *Cleome droserifolia* were identically analysed as discussed above for the Ae-Lnc (Additional file 5: Figure S2). We identified nine Cleomaceae-specific LncRNA based on 84,967 transcripts for *T. hassleriana* and 54,332 transcripts of *C. droserifolia* with ≥50 % sequence similarity*.* These nine transcripts had an average ORF and transcript-length (±SD) of 181.5 bp (±7.78 bp) and 675.71 bp (±201.53 bp) respectively (Additional file 4: Table S3 for species specific lengths). According to the categorization mentioned above, these nine LncRNAs consist of two GATUs, four TUs encoding NATs and 3 putative LincRNas. We did not identify any putative microRNA precursors.

### Conservation by position of transcribed LncRNAs

To exclude conserved non-coding sequences (CNSs) and to support functionality we only considered LncRNAs that we detected as transcribed by at least one species.

**Fig. 2:**
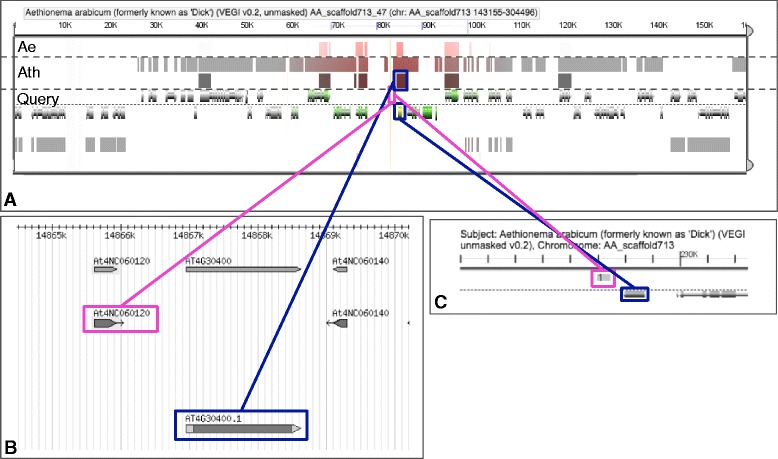
Example of collinearity and a positional conservation analysis of a Long non-coding RNA (LncRNA). **a** Screenshot from GeVo. GeVo calculates the collinearity of a query sequence with the genome of a subject organism. The query here is the nearest protein-coding gene of *Ae. arabicum* shown in (**c**), the subjects are *Ae. arabicum* and *A. thaliana.* Here there two collinear regions in *A. thaliana.* The position of the positionally conserved LncRNA is shown with a *pink* box, while the protein coding genes of *A. thaliana* and *Ae. arabicum* are shown with *blue* boxes. **b** Screenshot from the PLncDB website, shown are the *Arabidopsis thaliana* LncRNA (*pink*) and its nearest protein coding gene (*blue*). **c** Screenshot from the CoGe Blast HSP. *Pink* is the *Aethionema arabicum* transcript along the *Ae. arabicum* genome. *Blue* is the nearest *Ae. arabicum* protein coding gene. This SynFind and GeVo analyses can be redone with the following link: https://genomevolution.org/r/fmnf

**Fig. 3:**
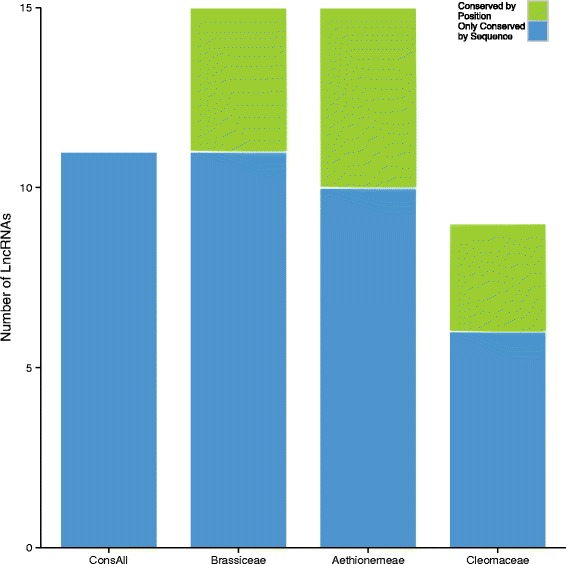
Bar-plot of the number of lineage-specific Long non-coding RNAs (LncRNAs). Every bar shows the total number of LncRNAs that are conserved by sequence within that clade. The *green* bars are the number of LncRNAs that are conserved by position across every clade and the *blue* bars are conserved by sequence within their lineage. For example: out of the nine LncRNAs that are by sequence conserved within the Cleomaceae, three are conserved by position in *Arabidopsis thaliana* and six are lineage specific by sequence and position to the Cleomaceae. ConsAll = LncRNA conserved by Brassicaceae, Cleomaceae and Aethionemeae

**Fig. 4:**
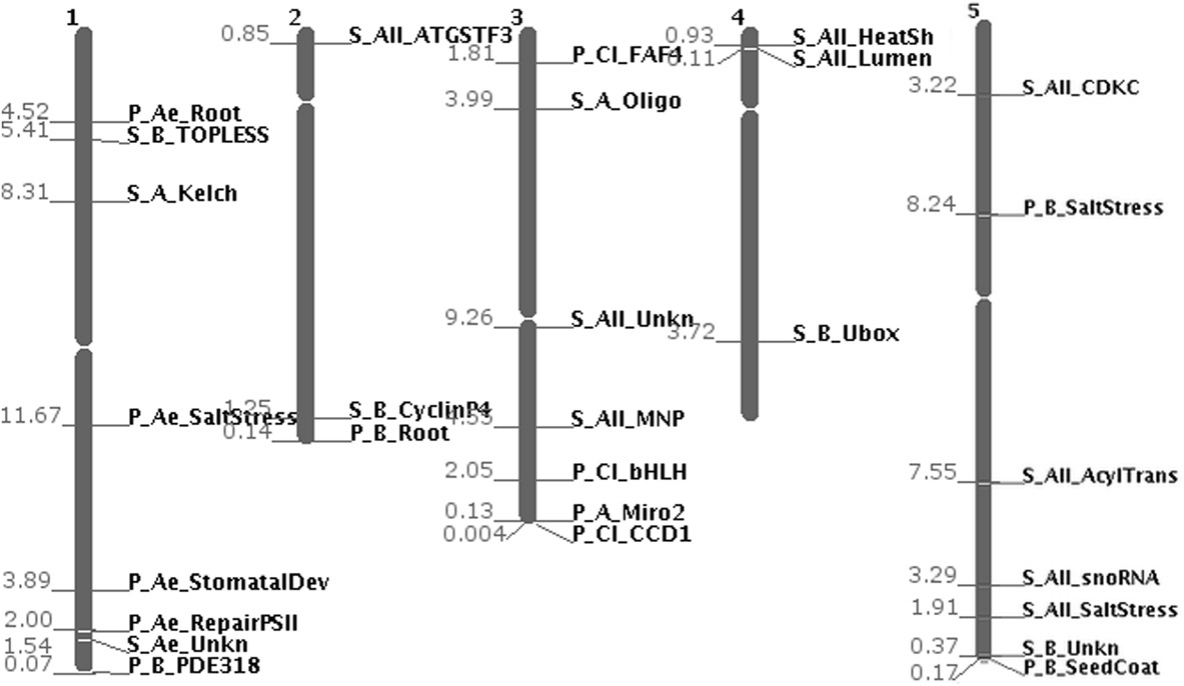
Distribution of the Long non-coding RNAs (LncRNAs) across the *Arabidopsis thaliana* genome. The positions are named as follow: conservation level_lineage of sequence conservation_gene function. Conservation level can be P: conserved by position across multiple lineages. S: only conserved by sequence and not by position. Ae: conserved by sequence only in Aethionemeae. All: conserved by sequence through Brassicaceae and Aethionemeae. B: conserved by sequence only in Brassicaceae, including Aethionemeae. Cl: conserved by sequence only in Cleomaceae. The numbers left of the chromosome are the distances from the gene to the end of the chromosome in Mega bases

We analysed the transcribed lineage-specific LncRNAs per clade and whether or not they are conserved by position within the genome of another lineages. Positional conservation was assessed with the CoGe-tools CoGeBlast, SynFind and GeVo ([21], see Methods for details). Out of the 39 LncRNAs that seemed to be lineage-specific at the nucleotide level (e.g. highly diverged between clades; 15 Bras-Lncs, 15 Ae-Lncs and 9 Cleo-Lncs) twelve were conserved by position in at least one of the other lineages (see Fig. 2 for an example and Additional file 8: Figure S3–S9 for the others). Depending on the clade (Aethionemeae specific, Cleomaceae specific or Brassicaceae specific) the percentage of LncRNAs that are not conserved by sequence but are conserved by position in another clade varied between 26 %-33 % (Fig. 3 and Additional file 7: Table S2). Figure 4 shows the distribution of the positionally conserved LncRNAs as positioned in the *A. thaliana* genome. Remarkably 66.66 % (8 out of 12) of the positionally conserved LncRNAs are within 2.5 MB from the chromosome ends, including in the subtelomeric regions (Fig. 4 and Additional file 7: Table S2). This corresponds with the finding of others that the telomeres and subtelomeric regions, have a higher gene density than the genomic average [26]. This could accordingly indicate the high number of gene regulatory elements.

Table 1 shows the functions of the neighbouring genes to the positionally conserved LncRNAs. The neighbouring genes of BrassLnc and Ae-Lnc (AT5G62420, AT5G24270 and AT1G50640) are associated with response(s) to salt stress. The *A. thaliana* genes neighbouring the positionally conserved Brass-Lnc and Ae-Lnc are involved at different levels of morphological and physiological development. These range from influencing root growth, to the development of stomata, to repairing photosystem II, to embryogenesis and mitochondrial morphogenesis (Table 1).

**Table 1:** Function of the nearest protein coding gene in *Arabidopsis thaliana of* positionally conserved LncRNAs

Level of sequence conservation	Transcribed in	Abbreviation	*A. thaliana* LncRNA	*A. thaliana* gene	Function	Reference
Brass. Specific	Ath	Bras_PDE318	At1NC112890	AT1G80770	Pigment defective 318 (PDE318)	
Brass. Specific	Ath	Bras_Root	At2NC078030	AT2G47750	Morphological Effect: Root Growth	[44]
					Encodes GH3.9, a member of the GH3 family auxin-responsive genes. gh3.9-1 mutants had greater primary root length.	
Brass. Specific	Ath	Bras_SaltStress	At5NC030470	AT5G24270	Response To Salt Stress	[45]
					Encodes a calcium sensor that is essential for K+ nutrition, K+/Na + selectivity, and salt tolerance	
Brass. Specific	Ath	Bras_SeedCoat	At5NC103231	AT5G67180	Morphological Effect: Seed Coat Mucilage	[46]
					Target of early activation tagged (EAT) 3 (TOE3)	
Ae. Specific	Ae	Ae_NuclStruc	At1NC016180	AT1G13230	Required for growth promotion and enhanced seed production mediated by the endophytic fungus Piriformospora indica in Arabidopsis.	
Ae. Specific	Ae	Ae_SaltStress	At1NC070280	AT1G50640	Response To Salt Stress and Involved in Leaf Senescence. Ethylene Responsive Element Binding Factor 3 (ERF3)	[47, 48]
Ae. Specific	Ae	Ae_StomatalDev	At1NC099220	AT1G70410	Morphological Efffect: Stomatal Development	[49]
					Beta Carbonic Anhydrase 4 (BCA4)	
Ae. Specific	Ae	Ae_RepairPSII	Group1797	AT1G75690	Physiological Effect: Repair of Photosystem II	[50]
					Low Quantum Yield of Photosystem II 1 (LQY1)	
Ae. Specific	Ae	Ae_Miro2	Group4790	AT3G63150	Physiological Effect: Embryo Genesis and Mitochondrial Morphogenesis. Miro-Related GTP-ASE 2 (MIRO2)	[51]
Cleo. Specific	Cleo	Cleo_bHLH	Group4645	AT3G57800	Basic helix-loop-helix (bHLH) DNA-binding superfamily protein. Transcription Factor Family	[52]
Cleo. Specific	Cleo	Cleo_Unknown	Seed_Group2679	AT3G06020	Regulation of shootmeristem size (FAF4).	[53]
Cleo. Specific	Cleo	Cleo_CCD1	Group4801	AT3G63520	Specifically expressed in vascular tissue.	[54]
					Carotenoid Cleavage Dioxygenase 1 (CCD1)	

‘Level of Sequence conservation’ denotes the level of lineage specificity of the LcnRNA at the nucleotide level

*Ae. Specific* Aethionemeae Specific, *Brass. Specific* Brassicaceae (including Aehtionemeae specific), *Cleo Specific* Cleomaceae specific, *Ae* Aethionemeae, *Ath Arabidopsis thaliana*, *Cleo* Cleomaceae

**Fig. 5:**
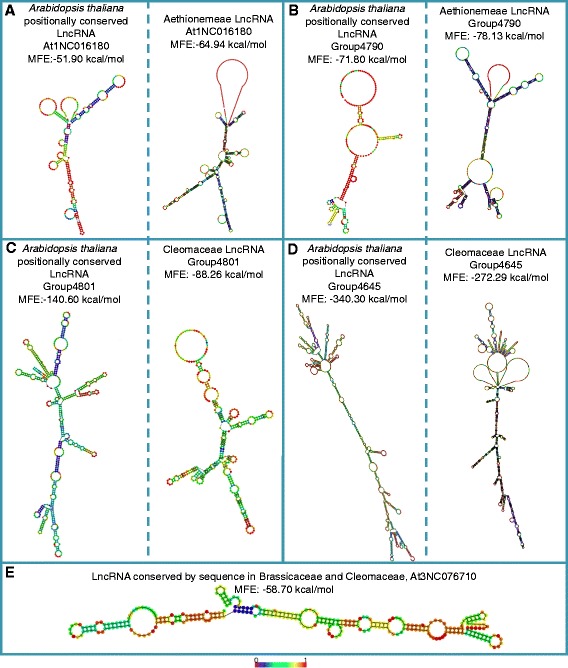
Secondary structures and Minimum Free Energy (MFE) of sequence and/or positionally conserved Long non-coding RNAs (LncRNAs). **a** LncRNAs that have both sequence conversation and positional conservation between Arabidopsis (*left*) and Aethionema (*right*) (**b**) LncRNAs that have only positional conservation between Arabidopsis (*left*) and Aethionema (*right*) (**c** LncRNAs that have both sequence conversation and positional conservation between Arabidopsis (*left*) and Tarenaya (*right*) (**d**) LncRNAs that have only positional conservation between Arabidopsis (*left*) and Tarenaya (*right*) (**e**) The LncRNA conserved by sequence and position in A. thaliana, *Aethionemea arabicum* and Cleomaceae*.* The colored bar below shows the baseparing probability for every structure

Some LncRNAs have been shown to have a stem-loop secondary structure [9, 27, 28]. We looked whether our positionally conserved LncRNAs have putative stable secondary structures and whether or not there are common features between the positionally conserved LncRNA (Fig. 5 and Additional file 9: Figure S10). The stability of a secondary structure is determined by its Minimum Free Energy (MFE), assuming that the lower the energy, the more stable the structure is [29]. Hence we regard structures with a MFE ≥ −80 kcal/mol as unstable. The secondary structures of the Ae-Lnc and their Ath-Linc counterparts are hence unstable (Fig. 5). The two Cleo-Linc and the Bras-Linc are more stable (Fig. 5). In accordance to the secondary structures found with other LncRNAs [9, 27, 28] all the stable structures have long stems and big loops on one side (Fig. 5).

## Discussion

As more complete genomes become available, it is possible to use genetic collinearity in addition to sequence similarity to address questions of conservation of noncoding sequences in a phylogenomic context. Using a comparative approach with the sister families Brassicaceae and Cleomaceae, we found LncRNAs are positionally conserved and expressed, but highly diverged at the nucleotide level. Hence here we found plant LncRNAs that are conserved by position but not by sequence, while the LncRNAs that are conserved by sequence are not conserved by position. While this result has been described earlier in comparative animal studies [30], to the best of our knowledge our work represents the first example of this trend in plants.

Long (intergenic) non-coding RNAs have been shown to affect the expression of their neighbouring genes [30], thus suggesting the importance of positional conservation in properly regulating adjacent genes encoding various traits. For example the positionally conserved LncRNAs found here are adjacent to genes involved in: response to salt stress, affecting important physiological functions (e.g. Photosystem II repair mechanism) or influencing morphological structures (e.g. root growth).

We based our analysis of positional conservation on the latest available genomes of *Aethionema arabicum, Tarenaya hassleriana* and *Arabidopsis thaliana.* The latest published *Aethionema arabicum* genome is >85 % of its total genome size [18] and the latest published genome of *Tarenaya hassleriana* is >94 % of its total genome size [19]. Although these genomes have already been published our analyses are always limited by quality of the genome assembly.

Long non-coding RNAs are a potentially important feature of gene regulation and genomes of eukaryotic organisms. To date, research into LncRNAs is more extensive in vertebrates than plants. Twenty-five out of the forty-eight functionally verified vertebrate LncRNAs have been conserved between human and mouse at >50 % sequence similarity [31]. Liu et al. [5], whose data has been explored here, found that <2 % of all the putative LncRNAs they found in *A. thaliana* are conserved across the plant kingdom. A similar number has been found by comparing maize (monocot) LncRNAs and *A. thaliana* (eudicot) [12]. The LncRNAs of legumes show only 5 % sequence conservation in non-legume plants [32]. A much higher percentage of the *Zea mays* LncRNAs, <25 %, are conserved in the closely related species sorghum [12]. Here we found that out of a total of 39 transcribed LncRNAs that are diverged at the nucleotide level, twelve are conserved by position. This is more than 30 % of the LncRNAs that we found in the transcriptomes of Aethionemeae and Cleomaceae.

Studies that take the position of LncRNAs into account primarily assume sequence conservation and additionally analyse whether or not those LncRNAs are also conserved by position. However in a comparison between zebra fish and humans Batista and Chang (2013) [30] found that LncRNAs with weak sequence conservation can still be fully functional, because they are still structurally and positionally conserved. Here we show similar results in plants: positional conservation of LncRNAs with weak sequence similarity between distantly related species.

The lack of sequence conservation but the presence of positional conservation might be explained by an increase in mutation rate for these regulatory elements. This has already been pointed out by Pang et al. [31], who hypothesized, for miRNAs and longer non-coding RNAs, that the type of interaction within a regulatory network can be under selection pressure rather than the sequence of the regulatory element itself. This hypothesis would fit well with the regulatory function and the position of LncRNAs. As LncRNAs regulate the expression of their neighbouring protein coding gene their interaction with this gene, and hence their position, rather than their sequence can be under selection.

We compared the secondary structure of the positionally conserved LncRNAs (Fig. 5). In addition to the positional conservation of LncRNAs their secondary structure might also be conserved. The Aethionemeae positionally conserved LncRNAs are less stable (higher MFE) than the Cleomaceae positionally conserved. A similar difference in stability is seen in their positionally conserved counterparts in *Arabidopsis thaliana.* The stability of the LncRNA secondary structure might be a step to subdivide the big group of LncRNAs.

Genomic regions of different species can be similar in sequence and can be completely collinear. However it is not a necessity that these sequences should be transcribed (see Additional file 7: Table S2). Here we used polyadenylated mRNAs to try to assess conservation of LncRNAs between different species. It has been shown that although LncRNAs can be polyadenylated, they are not always polyadenylated [33]. Consequently the positionally conservation shows only a subset of the plants possible transcripts. Moreover we applied the stringent rule that every LncRNA had to be transcribed in at least two species from the same lineage. Hence these results in a set of highly confident positional conserved LncRNAs that represent only the tip of the iceberg.

The small number of conserved LncRNAs found here is in accordance with the findings in other systems as discussed above [5, 12, 32]. The consistent finding of low nucleotide conservation raises new questions about the mutation rate of LncRNAs. Studies have shown that the mutation rate of LncRNAs resembles those of introns [12, 30, 31, 34], which could partially explain the lack of sequence similarity between LncRNAs over deep evolutionary time. However, this lack of sequence similarity did not result in a lack of conservation by position, which could indicate a conservation of function as it has been shown earlier that positional conservation also accounts for functional conservation [15, 30].

The presence of more than 65 % of the positionally conserved LncRNAs only within the 2.5 Mb of chromosome arms is remarkable and unexpected. In many plants the sub-telomeric regions consist of repeats, called satellites though these are absent in *A. thaliana* [26]. Their presence varies between species and even individuals within a species [26]. The satellites in the sub-telomeric region typically consist of large A-T rich repeat stretches, which makes bending the DNA easier and the heterochromatin formation tighter, which is shown by the presence of dense heterochromatin blocks [26, 35]. One suggested function of the presence of these satellite arrays is their support of the chromatin states in the sub-telomeric region [26]. However the absence of satellite arrays in *A. thaliana* might be compensated by the presence of LncRNAs that regulate the chromatin signatures of the protein coding genes in the sub-telomeric regions. We do not know of a specific reason why positionally conserved lncRNAs should be found only at chromosome ends. Certainly more research is needed to address this finding and the hypothesis stated above.

Preferably we would have tested whether the positionally conserved LncRNAs are also within 2.5 Mb of the chromosome arms of *Aethionema arabicum* and/or *Tarenaya hassleriana*. However chromosomal-level genome assemblies of these species are not available yet. However, we are working on these genome assemblies so that we can address these questions in the near future.

Long (intergenic) non-coding RNAs have been identified by investigating the deleterious effects of knocking out these conserved sequences on various traits, e.g. flowering time, fertility, etc. [6, 8, 9]. These wet-lab experiments are crucial to understand the functionality of any putative pathway (from gene and transcription to fitness effects). They can confirm the lack of small ORFs in LncRNAs and understand the full pathway on which the LncRNA has an effect, whether that is on neighbouring genes or across chromosomes [30, 36].

## Conclusion

To summarize, we have shown here, using the Brassicaceae and Cleomaceae phylogenomic system that transcribed plant Long non-coding RNAs (LncRNAs) that seem to be only conserved within one lineage at the sequence level are conserved in other lineages at the same genomic position. The positional conservation could also imply a conservation of function but a divergence of sequence. Moreover, >65 % of the positionally conserved LncRNAs are located within 2.5 Mb of the telomeric region. This emphasizes the gene regulatory role that LncRNAs can have. These results imply that lineage specificity should not only be regarded at the nucleotide level but also at the positional level.

## Methods

### Transcriptome isolation, library preparation and assembly

*Aethionema arabicum, Ae. carneum, Ae. spinosa, Ae. grandiflorum, Tarenaya hassleriana* and *Cleome droserifolia* seeds were germinated in sowing soil and grown in the greenhouse at the University of Amsterdam (18 °C at night, 20 °C day temperature, 12 h light, 12 h dark). Table 2 shows the tissues used for RNA isolation. To decrease RNA degradation the tissues were ground in liquid nitrogen and RNA was immediately isolated using PureLink™ RNA mini kit (Ambion, Life Technologies Corporation, Carlsbad, CA, USA), followed by a DNase treatment using the TURBO DNA-free™ kit (Ambion), according to the manufacturers protocols. The RNA quality and quantity was checked on a 1 % agarose gel stained with ethidium-bromide in a 1x TBE buffer and on a NanoDrop 1000© spectrophotometer (Thermo Fisher Scientific, Wilmington, DE, USA). The samples were dried with GenTegraTM (GenVault, Carlsbad, CA, USA) for shipment to the Sequencing Core of the University of Missouri-Colombia. The ds-cDNA library was constructed following the manufacturers protocol of the TruSeq-RNATM kit (Illumina, San Diego, CA, USA). The six new transcriptomes used here were selected for mRNA during the cDNA synthesis. Thus all the non-polyadenylated LncRNAs were not sequenced. *Aethionema grandiflorum* and *A. spinosa* were paired-end sequenced with the Illumina Hiseq2000 sequencer on 1x100bp lanes, with 3 lines per lane. The *Ae.arabicum* transcriptome was *de novo* assembled using Trininity [37]. The *Ae. carneum, Ae. grandiflorum* and *Ae. spinosa* transcriptomes were assembled against the *Ae. arabicum* contigs with NextGene V2.17® (SoftGenetics, State College, PA, USA) with matching requirements of ≥ 40 bp and ≥ 90 % similarity and ≤20 % present mutations. For each line a consensus sequence was constructed with the following parameter settings: 90 % minimum of aligned reads for homozygosity, 25 % as the cut-off for aligned read to be heterozygous and 85 % as the percentage of reads that are aligned for a homozygote indel.

**Table 2:** Species and tissues used for RNA isolation

Species	Tissues
*Aethionema arabicum & A. carneum*	Fruits, flowers, buds, apical meristem, leaves and side buds from fully grown plants, leaves and apical meristem from juvenile plants, and the whole seedling including the roots
*Aethionema grandiflorum & A. spinosa*	Meristem, leaves and young stems
*Tarenaya hassleriana*	Buds, open flowers and the apical meristem
*Cleome droserifolia*	Young leaves, roots and flowers

### Genomes, CDSs and LncRNA

The *Athionema arabicum* and *Tarenaya hassleriana* genomes were downloaded from the CoGe Website [19]. The CDSs of *Brassica rapa, Arabidopsis lyrata* and *Eutrema halophila* come from the PlantGDB website [38] and the *Arabidopsis thaliana* (Ath) CDS v10 from TAIR [39]. The proteomes of *Zea* mays, *Oryza sativa, Brachypodium distachion, Sorghum bicolore* and *Sorghum italica* were downloaded from Phytozome [40]. These latter CDS and proteomes were used in the OrthoMCL analysis (see Additional file 5: Figure S2) to ascertain that the LncRNAs are lineage specific. The location of Ath LncRNAs (Ath-Lnc) were downloaded from the PLncDB website [41] and used to extract the sequences from the *A. thaliana* chromosomes [39] with an in-house python script. All the genomes present in November 2013 in Phytozome [40] were downloaded for latter analyses.

### OrthoMCL, blast and positional conservation analyses

OrthoMCL [20], is based on reciprocal best blast hits (RBH) and uses a cluster algorithm (MCL) to cluster the RBHs. Depending on the Blast that is performed it is possible to use OrthoMCL with nucleotide or protein sequences. We used OrthoMCL with BlastN, query identity = 50 % and evalue = 1e-10, was used to assign orthologous groups to the lineage of interest (Additional file 1: Figure S1 and Additional file 10: Figure S11). All blasts were done with command-line blast [42] against the in-house made database of the Phytozome genomes and/or against the NCBI database with the ‘–remote’ command. The ORF size was assessed through the VirtualRibosome website [43] for all six frames and with a strict start codon. The location of the *Ae. arabicum* transcripts and *T. hassleriana* transcripts to the nearest genes on their own genomes was assessed with CoGeBlast [21]. The *Ae. arabicum* unmasked genome v2.5 and *T. hassleriana* unmasked genome V4 were used. Only when the transcripts had a query hit of ≥50 % and a HSP = 1 they were assumed to hit to the correct location on the genome. Alternative splicing was excluded by this assumption, as is also the case for redundant genomic hits. SynFind and GeVo [21] were used to assess collinearity between the region of nearest protein coding gene of the LncRNA and the *A. thaliana*, *Ae. arbicum* and/or *T. hassleriana* genome(s). For example: if a protein coding gene of Ae-Lnc was collinear with a region in *A. thaliana* the ‘GenomeBrowse’ utility of PLncDB [41] was used to assess whether there was a LncRNA in the same direction (upstream, downstream or as a natural antisense) that corresponds with the location of the Ae-Lnc (See Fig. 2). Hence these are LncRNAs different at the sequence level but are similar at position (see also Additional file 10: Figure S11 for an counter example).

All transcripts were tested to see whether or not they could be micro-RNA precursors. To this end, they were blasted (BlastN) against the mirBase database [44]. We used the RNAfold server [45] to see whether the transcripts could have a stable secondary structure as a microRNA. The structure was assumed to be stable if the Gibbs free energy was between −30 and −80 kcal/mol.

### Conserved LncRNA and secondary structure

The conservation of LncRNA was tested according to the pipeline as described in Additional file 1: Figure S1. This was done with the 10 %, 20 % and 50 % query identity for the OrthoMCL analyses at the beginning of the pipeline.

To assess whether the positionally conserved LncRNAs could have stable secondary structures the RNAalifold and RNAfold servers [45] were used. RNAalifold uses aligned sequences of more than two species, while RNAfold calculates secondary structures based on a singe RNA sequence. For the Ae-Lncs we used the transcripts of *Ae. arabicum, Ae. grandiflorum, Ae. carneum* and *Ae. spinosa.* Transcripts from the same species (if there present in the OrthoMCL analysis, see above) were used for the Brassicaceae specific LincRNAs. For the Cleomaceae specific LincRNAs of both *T. hassleriana* and *C. drosofolia* were used. To compare the positionally conserved LncRNAs the secondary structures of the Ath-Linc were also calculated.

## Additional files

Additional file 1: Figure S1. Pipeline to assess the transcribed (top-panel) and genomic (bottom-panel) Long non-coding RNAs (LncRNA) that are conserved at the nucleotide level throughout the Brassicaceae and Cleomaceae, or are specific to the Brassicaceae. (PDF 42 kb) Additional file 2: Table S1. Transcript and ORF length of *Tarenaya hassleriana* and Aethionemeae transcripts conserve by sequence. The sequence similarities percentages are cut-offs of sequence similarity within OrthoMCL. (DOCX 55 kb) Additional file 3:
**Long non-coding RNAs specific for the Brassicaceae, genomic.** (XLSX 41 kb) Additional file 4 :Table S3. Transcript and ORF length of Aethionemeae and Cleomaceae specific Long non-coding RNAs. (DOCX 39 kb) Additional file 5: Figure S2. Pipeline to assess the LncRNAs specific to *Aethionemeae* or Cleomaceae. (PDF 51 kb) Additional file 6: Table S4. Transcript and ORF length of Aethionemeae transcripts that are Brassicaceae specific. The sequence similarities percentages are cut-offs of sequence similarity within OrthoMCL. (DOCX 56 kb) Additional file 7: Table S2. Sheet 1 shows the transcript names of *Aethionema arabicum, Tarenaya hassleriana* and *Arabidopsis thaliana* and whether or not they are conserved by position or only by sequence across the different lineages. Sheet 2 shows the distances of the positionally conserved LncRNAs to the nearest end of *A. thaliana* chromosomes. (XLSX 22 kb) Additional file 8: Figure S3–S6. Analyses of collinearity and positional conservation of sequentially diversified Aethionemeae LncRNAs. (A) Screenshot from GeVo. GeVo calculates the collinearity of a query sequence with the genome of a subject organism. The query here is the nearest protein coding gene of *Ae. arabicum* shown in B, the subjects are *Ae. arabicum* and *A. thaliana.* The position of the positionally conserved LncRNA is shown with a pink box, while the protein coding genes of *A. thaliana* and *Ae. arabicum* are shown with blue boxes. (B) Screenshot from the PLncDB website, shown are the *Arabidopsis thaliana* LncRNA (pink) and its nearest protein coding gene (blue). (C) Screenshot from the CoGe Blast HSP. Pink is the *Aethionema arabicum* transcript along the *Ae. arabicum* genome. Blue is the nearest *Ae. arabicum* protein coding gene. **Figure S7–S9.** Analyses of collinearity and positional conservation of sequentially diversified Cleomaceae LncRNAs. (A) Screenshot from GeVo. GeVo calculates the collinearity of a query sequence with the genome of a subject organism. The query here is the nearest protein coding gene of *Taranaya hassleriana*, the subjects are *T. hassleriana* and *A. thaliana.* The position of the positionally conserved LncRNA is shown with a pink box, while the protein coding genes of *A. thaliana* and *T. hassleriana* are shown with blue boxes. (B) Screenshot from the PLncDB website, shown are the *Arabidopsis thaliana* LncRNA (pink) and its nearest protein coding gene (blue). (ZIP 716 kb) Additional file 9: Figure S10. Secondary structures and Minimum Free Energy (MFE) of sequence and/or positionally conserved LncRNAs. (A) LncRNAs that have both sequence conversation and positional conservation between Arabidopsis (left) and Aethionema (right) (B) LncRNAs that have only positional conservation between Arabidopsis (left) and Aethionema (right) (C) LncRNAs that have both sequence conversation and positional conservation between Arabidopsis (left) and Tarenaya (right) (D) LncRNAs that have only positional conservation between Arabidopsis (left) and Tarenaya (right) (E). The colored bar below shows the baseparing probability for every structure. (PDF 667 kb) Additional file 10: Figure S11. Example of an analysis of collinearity and no positionally conservation of a sequentially conserved LncRNA. The example is a LncRNA conserved at the sequence level within the Brassicaceae. A) Screenshot from the PLncDB website, shown are the *Arabidopsis thaliana* LncRNA (green) and its nearest protein coding gene (blue). B) Screenshot from the CoGe Blast HSP. Green is the *Aethionema arabicum* transcript along the *Ae. arabicum* genome. Blue is the nearest *Ae. arabicum* protein coding gene. C) Screenshot from GeVo. GeVo calculates the collinearity of a query sequence with the genome of a subject organism. The query here is the nearest protein coding gene of *Ae. arabicum* shown in B, the subjects are *Ae. arabicum* and *A. thaliana.* The query here shows two collinear regions in *A. thaliana.* The position of LncRNA is shown with a green box, while the protein coding genes of *A. thaliana* and *Ae. arabicum* are shown with blue boxes. D) Zoom in of the *A. thaliana* region that is collinear with *Ae. arabicum* and corresponds with the nearest *A. thaliana* nearest protein coding gene shown in A. These SynFind and GeVo analyses can be redone with the following link: https://genomevolution.org/r/fmqj. (PDF 146 kb)

## Abbreviations

LncRNA: Long non-coding RNA
LincRNA: Long intergenic non-coding RNA
Ath-Linc: Long intergenic non-coding RNA from [5]
Ae-Lnc: Long non-coding RNA conserved at nucleotide level by 4 Aethionemeae species
Brass-Lnc: Long non-coding RNA conserved at the nucleotide level by *Arabidopsis thaliana* and *Aethionema arabicum*
Cleo-Lnc: Long non-coding RNA conserved at the sequence level by two Cleomaceae species
All-Lnc: Long non-coding RNA conserved at the nucleotide level by the Brassicaceae and Cleomaceae
