# Supplementary figures and images for: Positionally-conserved but sequence-diverged: identification of long non-coding RNAs in the Brassicaceae and Cleomaceae
Source: BMC Plant Biol. 2015 Sep 11;15:217. doi: 10.1186/s12870-015-0603-5 (PMC4566204; doi:10.1186/s12870-015-0603-5)

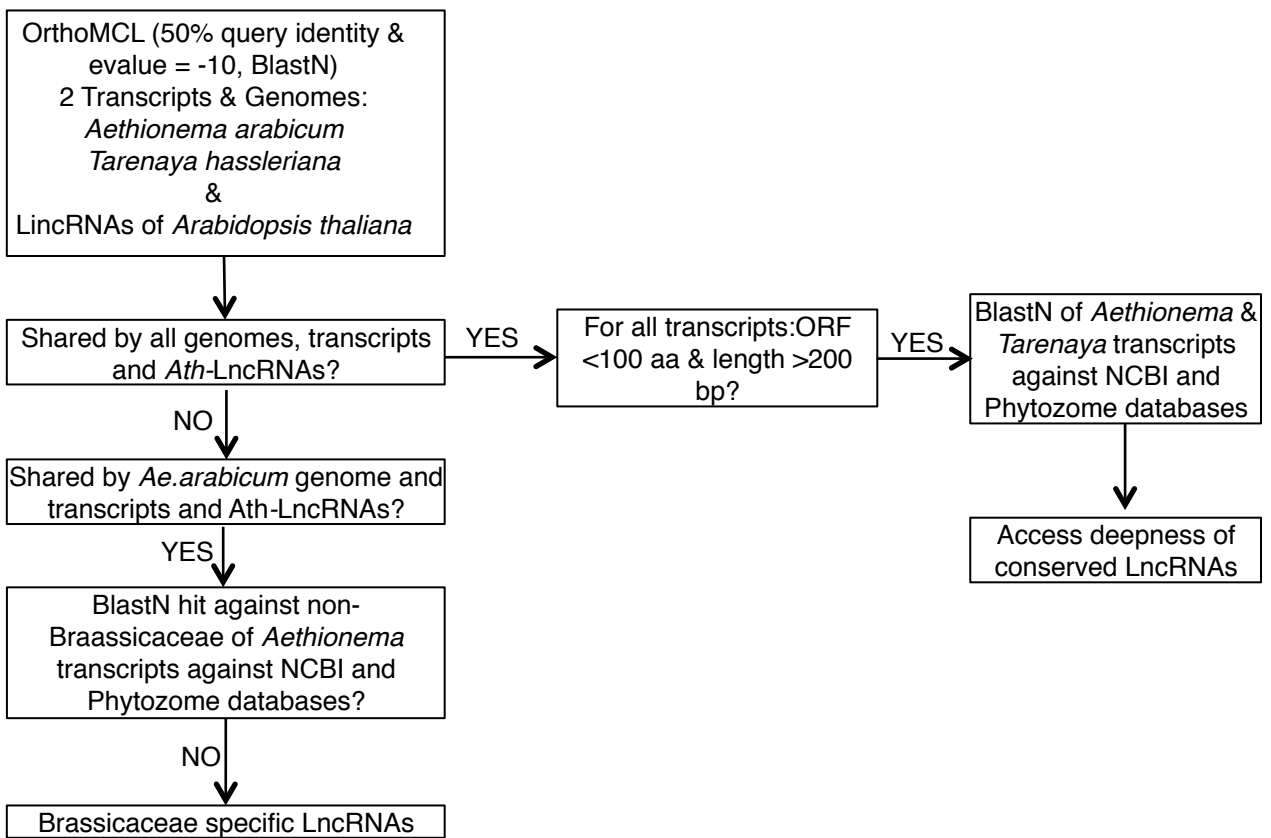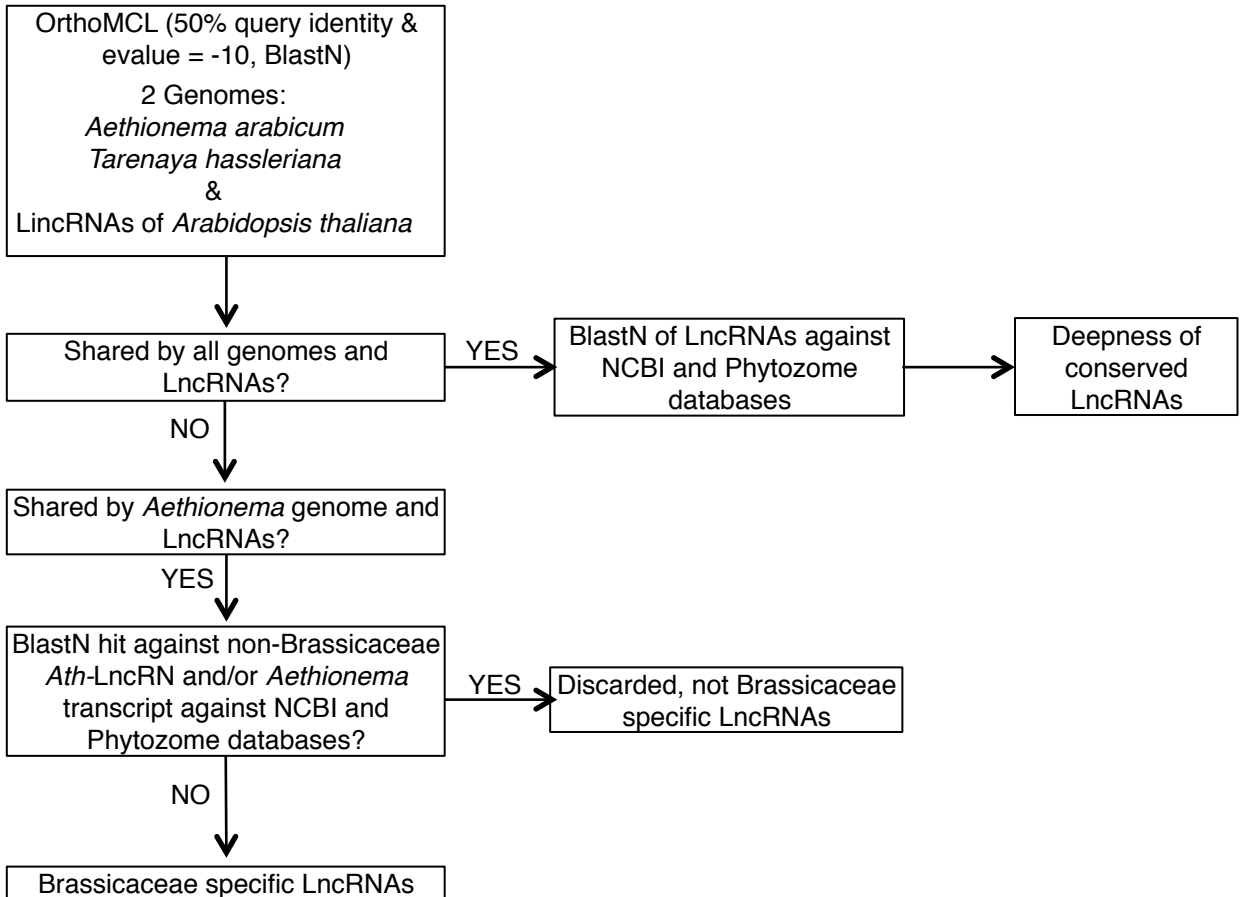

Supplement: Additional file 1: Figure S1. — Pipeline to assess the transcribed (top-panel) and genomic (bottom-panel) Long non-coding RNAs (LncRNA) that are conserved at the nucleotide level throughout the Brassicaceae and Cleomaceae, or are specific to the Brassicaceae. (PDF 42 kb) [file 12870_2015_603_MOESM1_ESM.pdf]

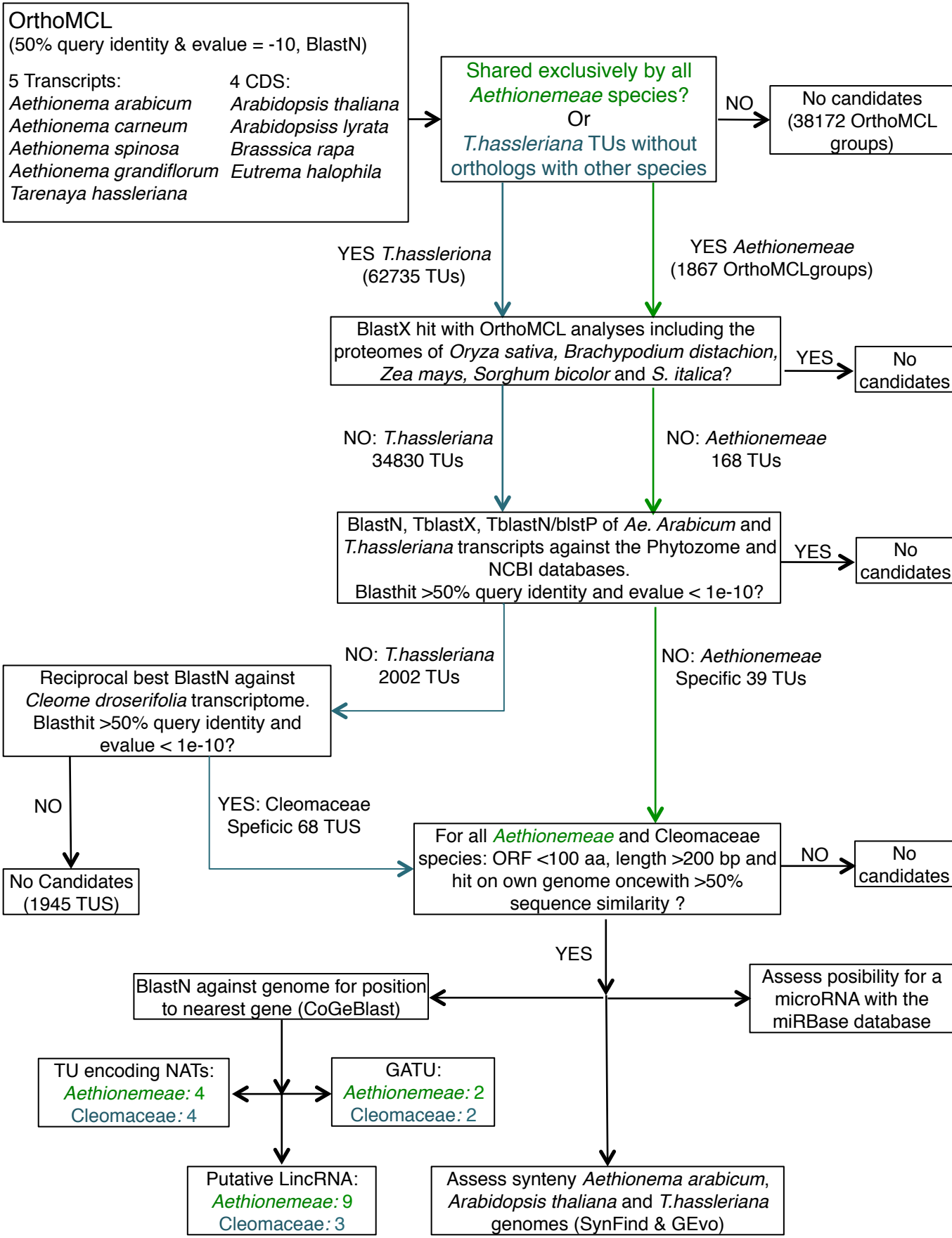

Supplement: Additional file 5: Figure S2. — Pipeline to assess the LncRNAs specific to Aethionemeae or Cleomaceae. (PDF 51 kb) [file 12870_2015_603_MOESM5_ESM.pdf]

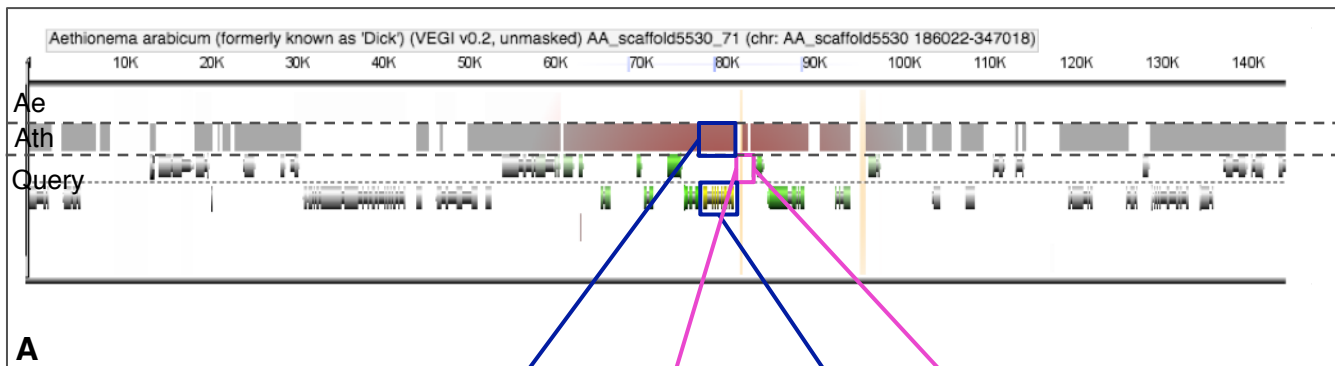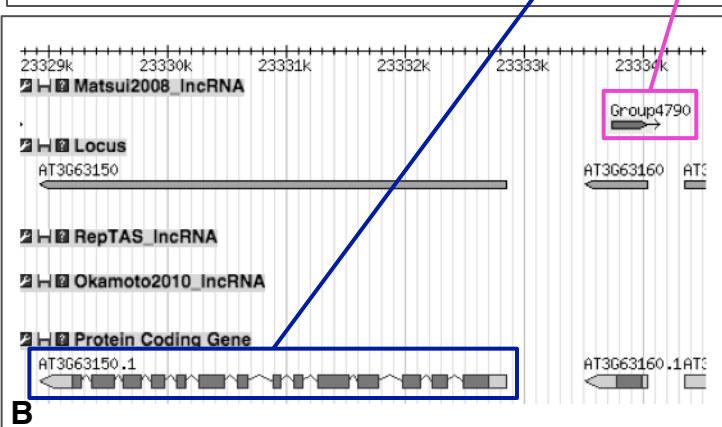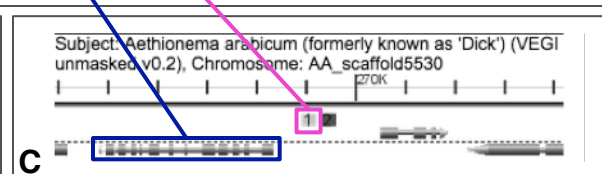

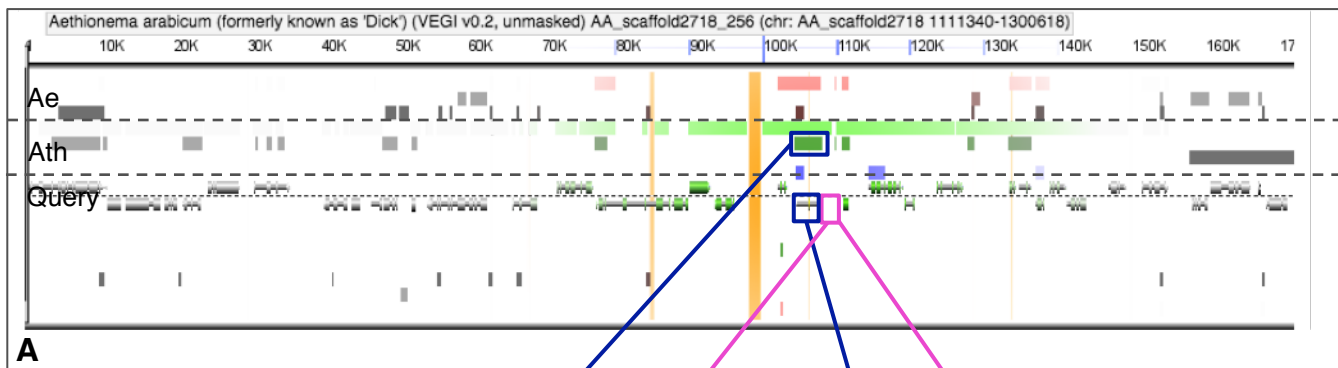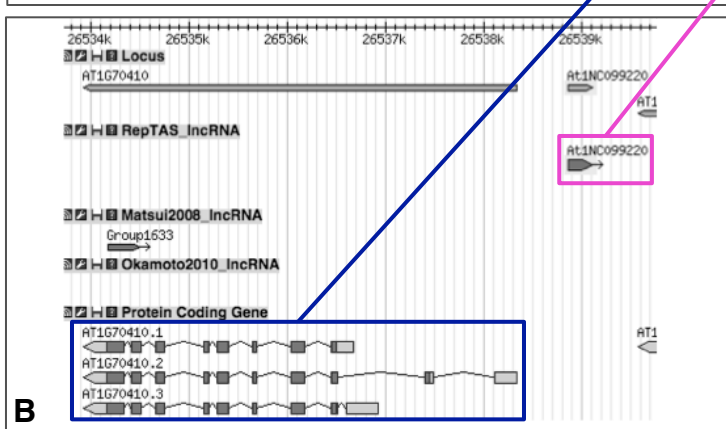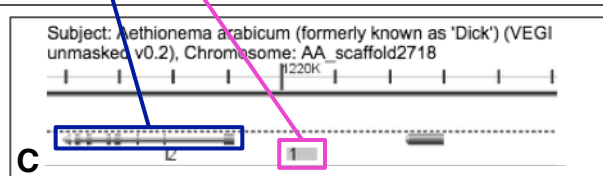



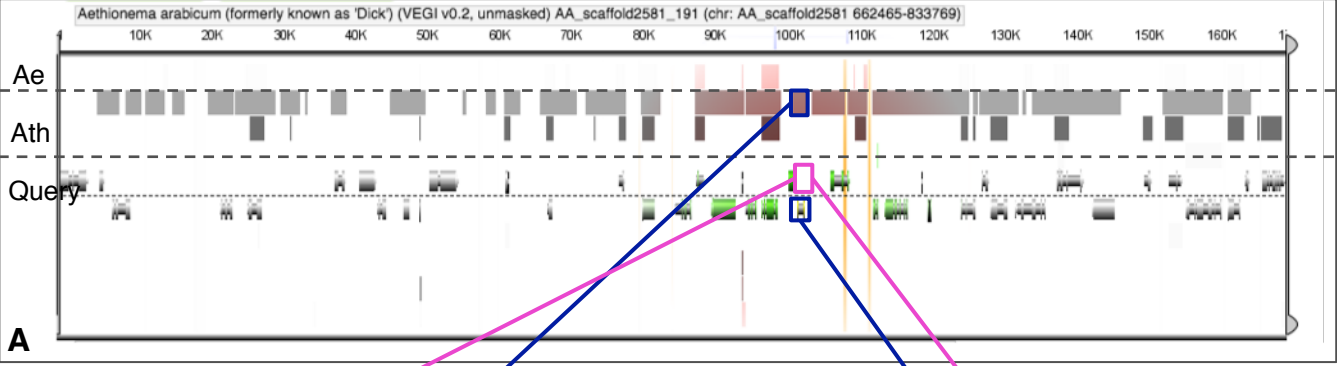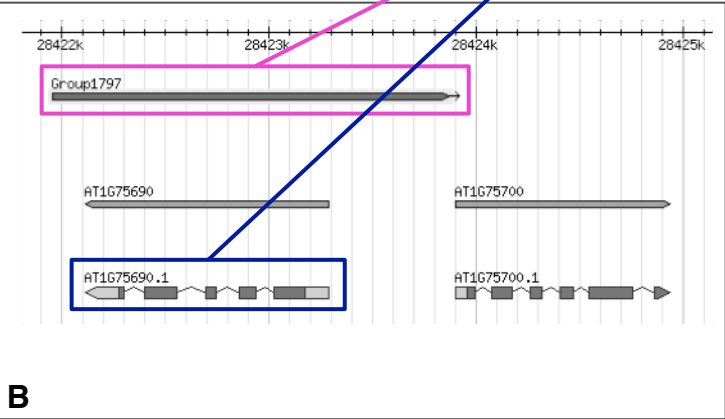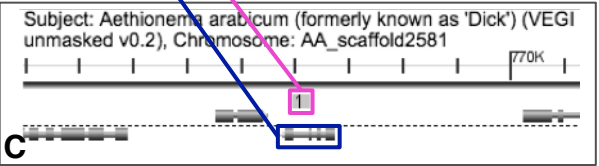

Supplement: Additional file 8: Figure S3–S6. — Analyses of collinearity and positional conservation of sequentially diversified Aethionemeae LncRNAs. (A) Screenshot from GeVo. GeVo calculates the collinearity of a query sequence with the genome of a subject organism. The query here is the nearest protein coding gene of Ae. arabicum shown in B, the subjects are Ae. arabicum and A. thaliana. The position of the positionally conserved LncRNA is shown with a pink box, while the protein coding genes of A. thaliana and Ae. arabicum are shown with blue boxes. (B) Screenshot from the PLncDB website, shown are the Arabidopsis thaliana LncRNA (pink) and its nearest protein coding gene (blue). (C) Screenshot from the CoGe Blast HSP. Pink is the Aethionema arabicum transcript along the Ae. arabicum genome. Blue is the nearest Ae. arabicum protein coding gene. Figure S7–S9. Analyses of collinearity and positional conservation of sequentially diversified Cleomaceae LncRNAs. (A) Screenshot from GeVo. GeVo calculates the collinearity of a query sequence with the genome of a subject organism. The query here is the nearest protein coding gene of Taranaya hassleriana, the subjects are T. hassleriana and A. thaliana. The position of the positionally conserved LncRNA is shown with a pink box, while the protein coding genes of A. thaliana and T. hassleriana are shown with blue boxes. (B) Screenshot from the PLncDB website, shown are the Arabidopsis thaliana LncRNA (pink) and its nearest protein coding gene (blue). (ZIP 716 kb) [file 12870_2015_603_MOESM8_ESM.zip › add7/Additionalfigure3-6.pdf]

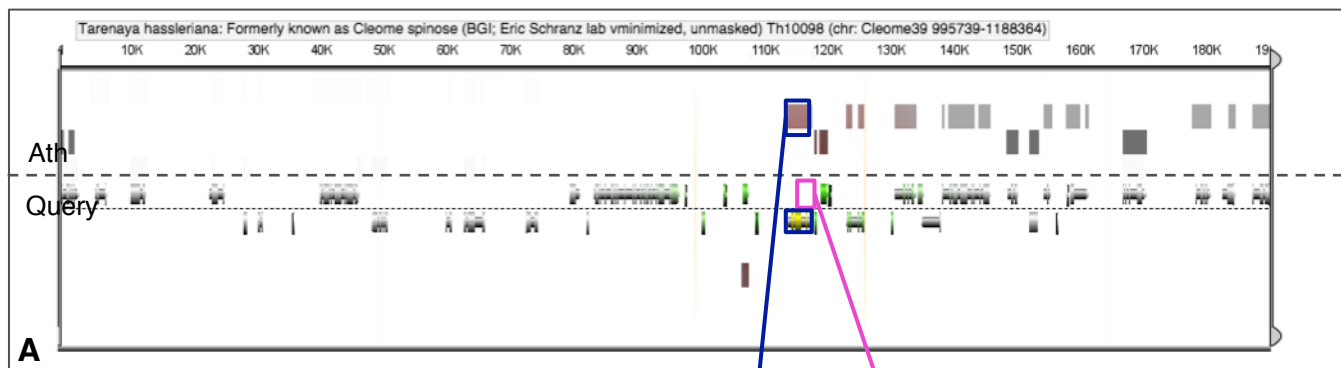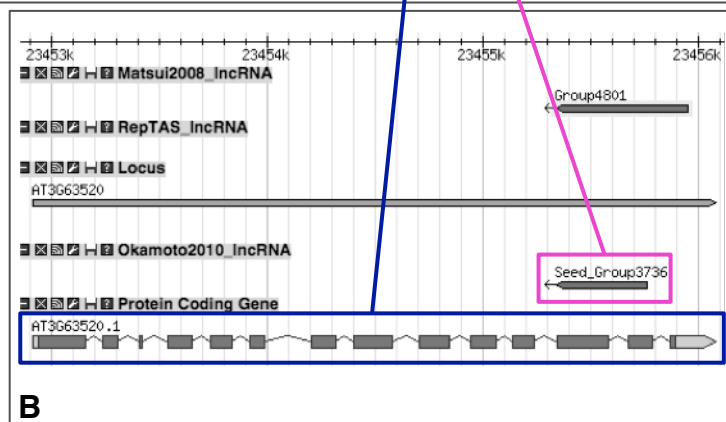

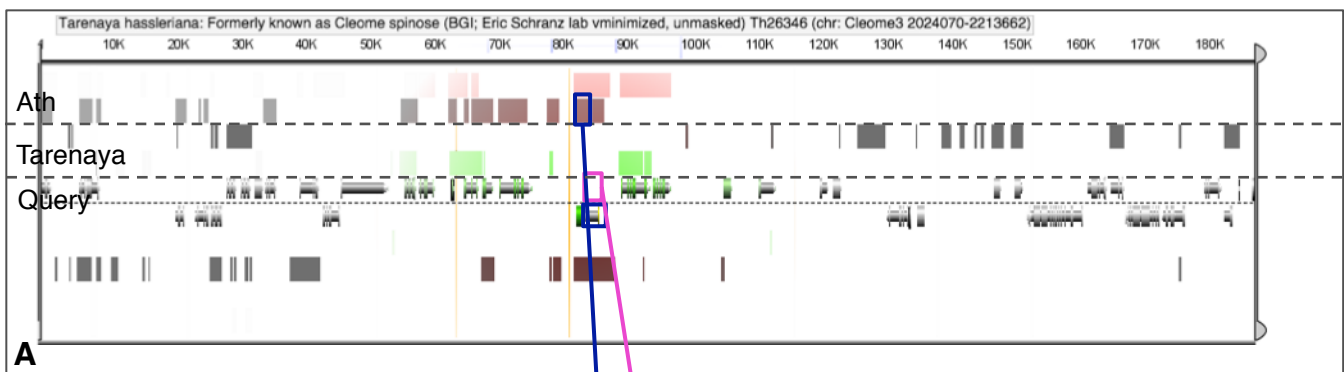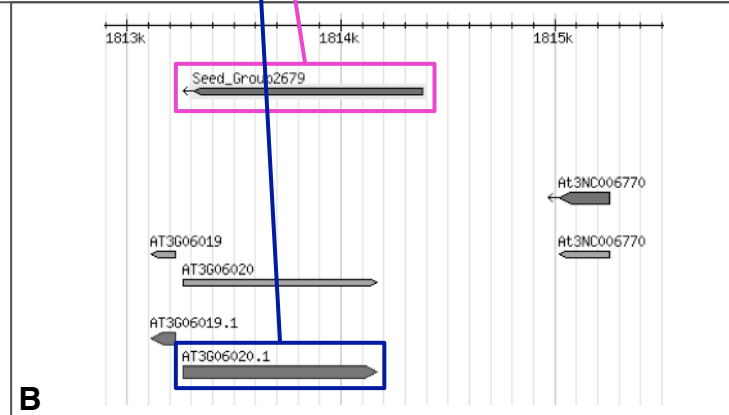

Supplement: Additional file 8: Figure S3–S6. — Analyses of collinearity and positional conservation of sequentially diversified Aethionemeae LncRNAs. (A) Screenshot from GeVo. GeVo calculates the collinearity of a query sequence with the genome of a subject organism. The query here is the nearest protein coding gene of Ae. arabicum shown in B, the subjects are Ae. arabicum and A. thaliana. The position of the positionally conserved LncRNA is shown with a pink box, while the protein coding genes of A. thaliana and Ae. arabicum are shown with blue boxes. (B) Screenshot from the PLncDB website, shown are the Arabidopsis thaliana LncRNA (pink) and its nearest protein coding gene (blue). (C) Screenshot from the CoGe Blast HSP. Pink is the Aethionema arabicum transcript along the Ae. arabicum genome. Blue is the nearest Ae. arabicum protein coding gene. Figure S7–S9. Analyses of collinearity and positional conservation of sequentially diversified Cleomaceae LncRNAs. (A) Screenshot from GeVo. GeVo calculates the collinearity of a query sequence with the genome of a subject organism. The query here is the nearest protein coding gene of Taranaya hassleriana, the subjects are T. hassleriana and A. thaliana. The position of the positionally conserved LncRNA is shown with a pink box, while the protein coding genes of A. thaliana and T. hassleriana are shown with blue boxes. (B) Screenshot from the PLncDB website, shown are the Arabidopsis thaliana LncRNA (pink) and its nearest protein coding gene (blue). (ZIP 716 kb) [file 12870_2015_603_MOESM8_ESM.zip › add7/AdditionalFigure7-9.pdf]

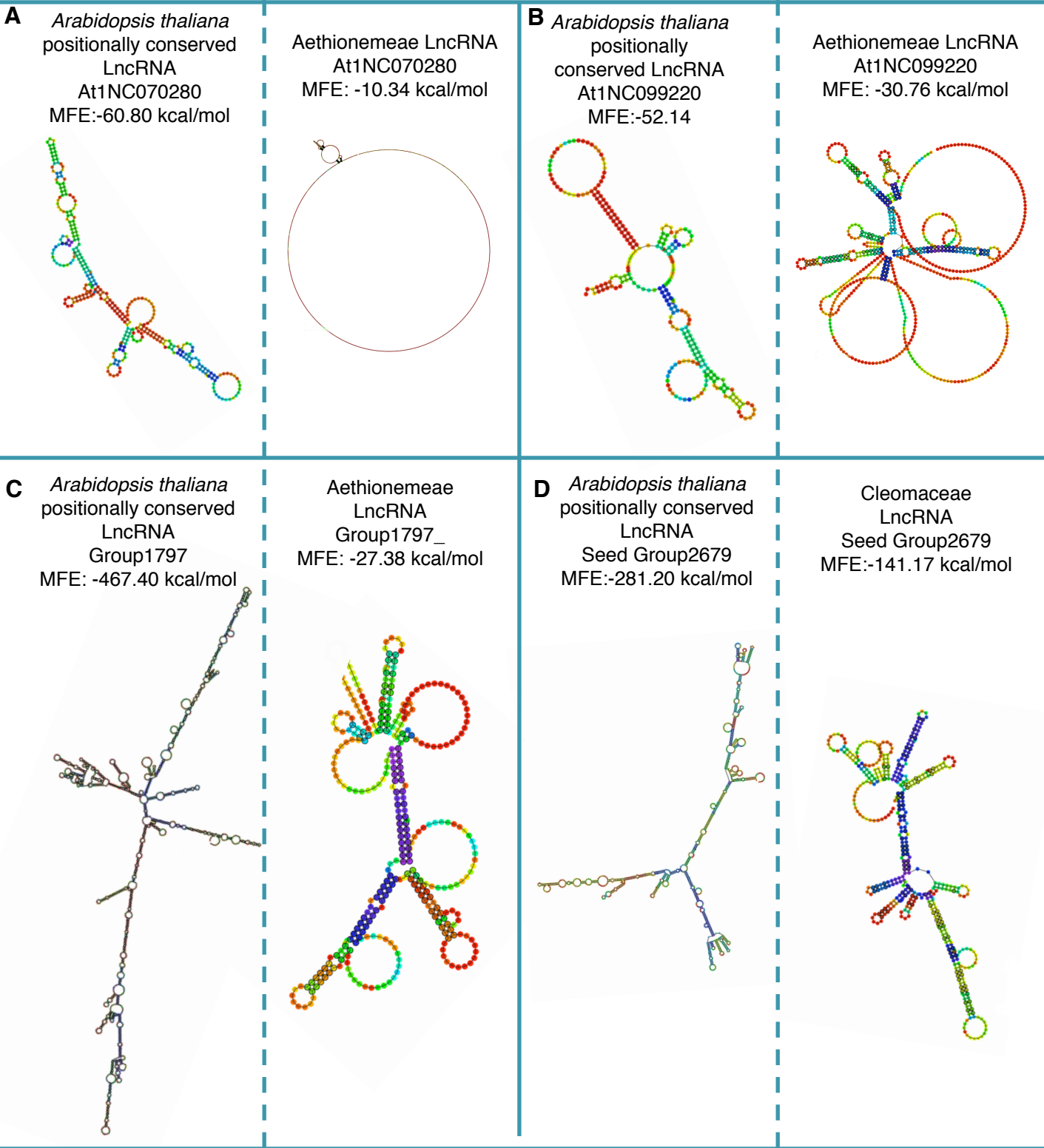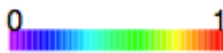

Supplement: Additional file 9: Figure S10. — Secondary structures and Minimum Free Energy (MFE) of sequence and/or positionally conserved LncRNAs. (A) LncRNAs that have both sequence conversation and positional conservation between Arabidopsis (left) and Aethionema (right) (B) LncRNAs that have only positional conservation between Arabidopsis (left) and Aethionema (right) (C) LncRNAs that have both sequence conversation and positional conservation between Arabidopsis (left) and Tarenaya (right) (D) LncRNAs that have only positional conservation between Arabidopsis (left) and Tarenaya (right) (E). The colored bar below shows the baseparing probability for every structure. (PDF 667 kb) [file 12870_2015_603_MOESM9_ESM.pdf]

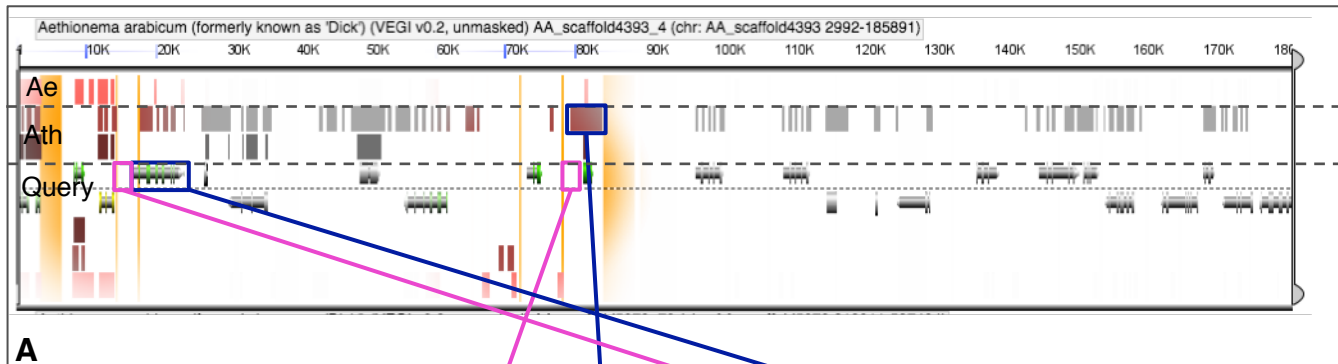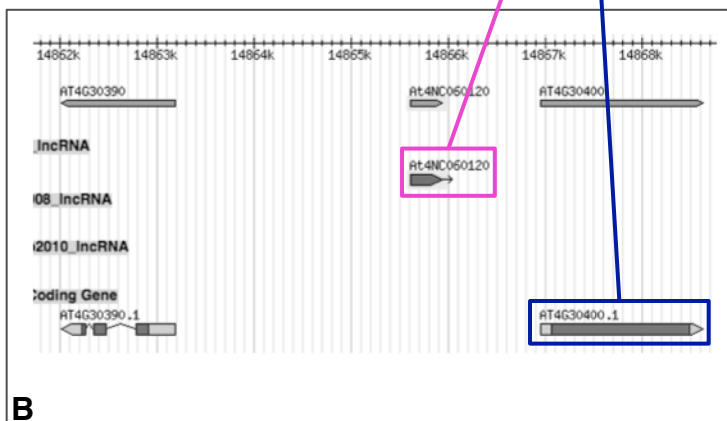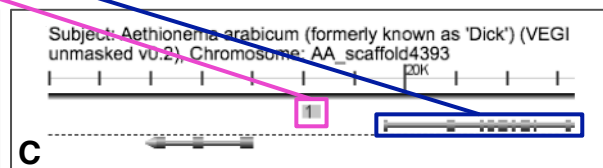

Supplement: Additional file 10: Figure S11. — Example of an analysis of collinearity and no positionally conservation of a sequentially conserved LncRNA. The example is a LncRNA conserved at the sequence level within the Brassicaceae. A) Screenshot from the PLncDB website, shown are the Arabidopsis thaliana LncRNA (green) and its nearest protein coding gene (blue). B) Screenshot from the CoGe Blast HSP. Green is the Aethionema arabicum transcript along the Ae. arabicum genome. Blue is the nearest Ae. arabicum protein coding gene. C) Screenshot from GeVo. GeVo calculates the collinearity of a query sequence with the genome of a subject organism. The query here is the nearest protein coding gene of Ae. arabicum shown in B, the subjects are Ae. arabicum and A. thaliana. The query here shows two collinear regions in A. thaliana. The position of LncRNA is shown with a green box, while the protein coding genes of A. thaliana and Ae. arabicum are shown with blue boxes. D) Zoom in of the A. thaliana region that is collinear with Ae. arabicum and corresponds with the nearest A. thaliana nearest protein coding gene shown in A. These SynFind and GeVo analyses can be redone with the following link: https://genomevolution.org/r/fmqj. (PDF 146 kb) [file 12870_2015_603_MOESM10_ESM.pdf]
