# Supplementary material for: Positionally-conserved but sequence-diverged: identification of long non-coding RNAs in the Brassicaceae and Cleomaceae
Source: BMC Plant Biol. 2015 Sep 11;15:217. doi: 10.1186/s12870-015-0603-5 (PMC4566204; doi:10.1186/s12870-015-0603-5)
Supplement: Additional file 2: Table S1. — Transcript and ORF length of Tarenaya hassleriana and Aethionemeae transcripts conserve by sequence. The sequence similarities percentages are cut-offs of sequence similarity within OrthoMCL. (DOCX 55 kb) [file 12870_2015_603_MOESM2_ESM.docx]

**Additional Table 1** Transcript and ORF length of *Tarenaya hassleriana* and Aethionemeae transcripts conserved by sequence.

| Sequence similarity  (%) | n | ORF length (bp)  (Average ± SD) | ORF range  (bp) | Transcript Length  (Average ± SD) | Transcript range  (bp) |
| --- | --- | --- | --- | --- | --- |
| *Tarenaya hassleriana* |  |  |  |  |  |
| 10% | 75 | 210.40 ± 50.45 | 96 - 297 | 854.53 ± 337.40 | 272 – 1933 |
| 20% | 69 | 208.91 ± 51.41 | 96 - 297 | 863.99 ± 342.26 | 272 – 1933 |
| 50% | 68 | 212.55 ± 50.18 | 96 - 297 | 846.65 ± 328.88 | 398 – 1933 |
|  |  |  |  |  |  |
| *Aethionema arabicum* |  |  |  |  |  |
| 10% and 20% | 2 | 189.00 ± 59.39 | 138 - 231 | 532.00 ± 234.76 | 366 – 698 |
| 50% | 12 | 189.25 ± 67.27 | 93 - 294 | 524.17 ± 167.54 | 371 – 975 |
