# Supplementary material for: Positionally-conserved but sequence-diverged: identification of long non-coding RNAs in the Brassicaceae and Cleomaceae
Source: BMC Plant Biol. 2015 Sep 11;15:217. doi: 10.1186/s12870-015-0603-5 (PMC4566204; doi:10.1186/s12870-015-0603-5)
Supplement: Additional file 4 :Table S3. — Transcript and ORF length of Aethionemeae and Cleomaceae specific Long non-coding RNAs. (DOCX 39 kb) [file 12870_2015_603_MOESM4_ESM.docx]

**Additional Table 4** Transcript and ORF length of Aethionemeae transcripts that are Brassicaceae specific.

| Sequence similarity (%) | n | ORF length (Average ± SD) | ORF  range (bp) | Transcript Length (Average ± SD) | Transcript range (bp) |
| --- | --- | --- | --- | --- | --- |
| 10% | 4 | 138.75 ± 68.03 | 81 - 213 | 458.75± 74.3 | 350 – 517 |
| 20% | 4 | 158 ± 68.69 | 81 - 213 | 439.3 ± 77.60 | 350 – 490 |
