# Supplementary material for: Positionally-conserved but sequence-diverged: identification of long non-coding RNAs in the Brassicaceae and Cleomaceae
Source: BMC Plant Biol. 2015 Sep 11;15:217. doi: 10.1186/s12870-015-0603-5 (PMC4566204; doi:10.1186/s12870-015-0603-5)
Supplement: Additional file 6: Table S4. — Transcript and ORF length of Aethionemeae transcripts that are Brassicaceae specific. The sequence similarities percentages are cut-offs of sequence similarity within OrthoMCL. (DOCX 56 kb) [file 12870_2015_603_MOESM6_ESM.docx]

**Additional Table 4** Transcript and ORF length of Aethionemeae and Cleomaceae specific Long non-coding RNAs.

| Species | n^a^ | ORF length ( bp)  (Average ± SD) | ORF  range (bp) | Transcript Length  bp (Average ± SD) | Transcript range (bp) |
| --- | --- | --- | --- | --- | --- |
| *A. arabicum* | 15 | 140.40 ± 44.95 | 81 - 234 | 523.87 ± 223.69 | 304 – 1233 |
| *A. carneum* | 16 | 155.25 ± 45.57 | 66 - 234 | 571.13 ± 251.89 | 218 – 1215 |
| *A. grandiflorum* | 15 | 134.60± 57.08 | 81 - 264 | 520.23 ± 223.67 | 304 – 1233 |
| *A. spinosa* | 20 | 153.30 ± 47.89 | 84 – 243 | 572.05 ± 254.98 | 204 – 1110 |
|  |  |  |  |  |  |
| *Tarenaya hassleriana* | 9 | 176.00±61.79 | 90-267 | 533.11±210.09 | 288-944 |
| *Cleome droserifolia* | 9 | 187.00±63.99 | 120-285 | 818.11±677.04 | 355-2079 |

^a^) number of transcript of every species that is orthologous to the other species from that lineage
